# Enhancing the conversational agent with an emotional support system for mental health digital therapeutics

**DOI:** 10.3389/fpsyt.2023.1148534

**Published:** 2023-04-17

**Authors:** Qing Wang, Shuyuan Peng, Zhiyuan Zha, Xue Han, Chao Deng, Lun Hu, Pengwei Hu

**Affiliations:** ^1^China Mobile Research Institute, Beijing, China; ^2^School of Information, Renmin University of China, Beijing, China; ^3^The Xinjiang Technical Institute of Physics and Chemistry, Chinese Academy of Sciences, Urumqi, China

**Keywords:** digital mental health, digital therapeutics, conversational agent, natural language processing, emotional support conversation

## Abstract

As psychological diseases become more prevalent and are identified as the leading cause of acquired disability, it is essential to assist people in improving their mental health. Digital therapeutics (DTx) has been widely studied to treat psychological diseases with the advantage of cost savings. Among the techniques of DTx, a conversational agent can interact with patients through natural language dialog and has become the most promising one. However, conversational agents' ability to accurately show emotional support (ES) limits their role in DTx solutions, especially in mental health support. One of the main reasons is that the prediction of emotional support systems does not extract effective information from historical dialog data and only depends on the data derived from one single-turn interaction with users. To address this issue, we propose a novel emotional support conversation agent called the STEF agent that generates more supportive responses based on a thorough view of past emotions. The proposed STEF agent consists of the emotional fusion mechanism and strategy tendency encoder. The emotional fusion mechanism focuses on capturing the subtle emotional changes throughout a conversation. The strategy tendency encoder aims at foreseeing strategy evolution through multi-source interactions and extracting latent strategy semantic embedding. Experimental results on the benchmark dataset ESConv demonstrate the effectiveness of the STEF agent compared with competitive baselines.

## 1. Introduction

Mental disorders have a higher lifetime prevalence and have a greater influence on people's quality-adjusted life expectancy (1). According to Organization (2), mental health issues such as depression affect more than 350 million people, which has been the leading cause of acquired disability. Without adequate treatment, a person suffering from mental health problems would get increasingly ill with multiple symptoms, such as insomnia and loss of interest. Therefore, it is vitally necessary to assist people in improving their mental health, given the prevalence of psychological diseases (3). While face-to-face psychological counseling is an effective approach to treating a variety of mental health issues, only a small percentage of individuals have access to it. According to Tong et al. (4), the demand for professional mental health therapists is high, and nearly 60 percent of those with a mental disorder are unable to receive treatment.

Due to the limited access to treatment and the increasing expenditures on healthcare, it is critical to develop digital health solutions (4, 5). Digital Therapeutics (DTx), a subset of digital health solutions, provides evidence-based therapeutic interventions. To prevent, manage, or treat a medical ailment, DTx leverages state-of-the-art artificial intelligence techniques to replace or enhance a variety of established psychological approaches to therapy (6). Artificial intelligence techniques have been widely employed in a variety of fields and have already been used in combination with drugs or other therapies to improve patient care and health outcomes (7–12).

DTx products are generally delivered via smartphones or computers, which offers patients more convenience and privacy. In particular, DTx products on smartphones can be multilingual. Thus, DTx has the potential to address the inadequacy of psychological treatment access. Patients suffering from major depressive disorder (MDD) frequently struggle to apply what they learn in therapy or lose motivation to do what their therapists assign them to do. DTx can help patients with MDD keep practicing their skills and improve their ability to move away from negative thoughts. At the same time, DTx can provide therapists with a wealth of additional information about their patients' daily lives. With the help of DTx, clinicians can adjust the treatment and communicate with patients online in real time, intervening as needed (13).

One of the most promising technologies for these DTx products is conversational agents. Conversational agents utilize natural language processing technologies to provide supplemental treatment or track adherence with patients. The advantages of conversational agents in mental health include giving people who require psychological counseling 24/7 access to treatment resources (13). Conversational agents can also inform patients about common therapeutic issues, remind patients about important therapeutic issues, and notify patients when the monitoring indicator value is out of range (14, 15).

Research shows that patients with severe symptoms are more likely to keep having a conversation with the conversational agent if they get emotional messages while they communicate (16, 17). However, because it may not be naturally possible to be able to express empathetically (18), many conversational agents are unable to fully understand the patient's individual needs, determine how the patient is feeling, and accurately show emotion in conversation. As a result, the role of conversation agents in DTx solutions is limited.

Introducing emotion into conversation systems has been widely studied since the early days. The emotional chatting machine (ECM) (19) was a noteworthy work in emotional conversation, capable of generating emotional responses based on pre-specified emotions and accurately expressing emotion in generated responses. Some works (20–24) concentrated on empathetic responding, which is good at understanding user emotions and responding appropriately, making responses more empathetic. Other works (25–27) learned to statistically predict the user's emotion using a coarse-grained conversation-level emotion label. However, accurate emotional expression and empathy are only the starting point of useful emotional support. Other skills should also consider other abilities.

Several emotion conversation datasets have also been built based on social context. Medeiros and Bosse (28), collected around 10,000 post-response pairs about stressful situations from Twitter, classified these tweets into different supportive categories, and collected supportive replies to them with crowd-sourcing workers. Based on the data, it was also determined which types of support were used most frequently and why. Sharma et al. (29) built an empathy conversation corpus of 10k (post-response) pairs with supporting evidence provided by the model using a RoBERTa-based bi-encoder model to identify empathy in conversations and extract rationales underlying its predictions. However, both prior datasets only contained single-turn conversations, which can only be used to support the exploration of simplified response scenarios with users at a coarse-grained emotion level.

To fully focus on the emotional support for conversational agents, the emotional support conversation (**ESC**) task was defined by Liu et al. (30). They also released the first large-scale multi-turn ESC dataset, **ESConv**, and designed an ESC framework. The ESC task aims at strategically comforting the user who wants to seek help to improve their bad emotional state; thus, the ESC framework has three stages (*Exploration, Comforting, and Action*). The first stage requires the supporter (or the conversational agent) to identify the user's problem, followed by properly selecting a support strategy to comfort the user for the second stage. Finally, the supporter should provide suggestions to evoke a positive mental state.

The ESC task, according to Liu et al. (30), has two fundamental problems. One of them is determining how to generate a strategy-constrained response with suitable strategy selection. Another challenge is how to dynamically model the user's mental state. Prior works on the ESC task mainly detect (31, 32) the interaction between the problem faced by the user and the user's present mental state. However, the user's mental state is complex and changes subtly throughout a conversation. An effective ES system should consider all mental states of the whole conversation. Identifying the user's fine-grained, dynamic mental state is critical in the multi-turn ESC scenario (15, 33). Moreover, some earlier works merely considered the dialog history to foresee the strategy and overlooked the past strategies the supporter used. Even though some of the past strategies may not have instantly alleviated users' distress, the past strategies are critical for having a long-term effect on reducing depression.

In this study, we propose the STEF agent, a novel emotional support conversation agent built on the ESC, to address the above issues. Our STEF agent is composed of an emotional fusion mechanism and a strategy tendency encoder. The emotional fusion mechanism focuses on capturing subtle emotional changes by combining the representation of historical and present mental states via a fusion layer. The strategy tendency encoder aims at extracting latent strategy text semantic embedding and discovering strategy tendency. Thereafter, we implement a strategy classifier to foresee the future support strategy. At last, STEF agent can generate more supportive responses with fine-grained historical emotional understanding and an appropriate support strategy. In the following sections, we will look into the details.

## 2. Methods and materials

**Figure 1:**
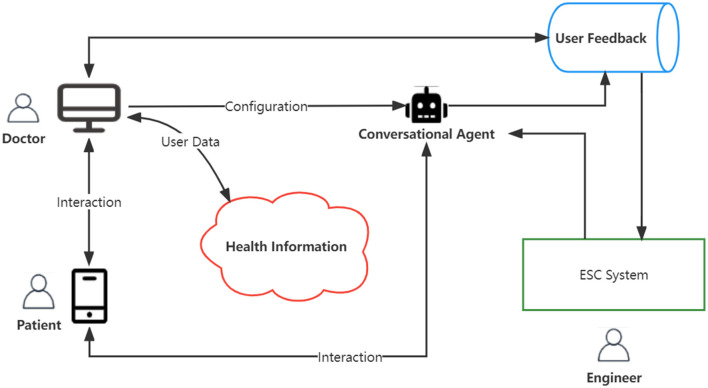
Digital therapeutics platform with the model of emotional support conversation.

In this section, we first introduce the ESC system on the digital therapeutic platform. As shown in Figure 1, the doctor can utilize the user data stored in the cloud service to personalize treatment and track the patient's compliance on the digital therapeutic platform. The conversation agent with the ability to provide emotional support can further comprehend the patient's situation and provide a considerate response or accurate medical advice based on the doctor's configuration and helping skills. Particularly in the mental health area, the conversation agent with this ability enables the agent to accompany the patient and act as a supervisor to avoid self-harm behaviors if necessary. The patient can have daily interactions with the conversation agent, and these interactions will be logged in the database as user feedback. Thereafter, the doctor obtains patients' feedback from the database to track the patient's treatment response and adjust therapy timely during the course of treatment. The engineer will employ patients' feedback to promote the performance of emotional support.

Our approach focused on promoting the performance of emotional support for conversation agents. We conducted our proposed model of ESC on the ESConv dataset. More details about the dataset are described in the next section *Emotional Support*. The construction of the ESC system is described in the section *STEF agent*.

### 2.1. Emotional support

The purpose of emotional support is to comfort seekers and provide suggestions to resolve the problems they face. Specifically, the emotional support conversation takes place between a seeker and a supporter, with the supporter attempting to gradually relieve the help seeker's distress and assist them in overcoming the challenges they confront as the conversation progresses. According to Tu et al. (31), it is not intuitive to provide emotional support, so conversational skills are critical for providing more support through dialog. Hence, the selection of a support strategy (conversational helping skills) in the ESC task is a significant challenge. Particularly, based on psychological research (34), choosing a appropriate support strategy is crucial for ensuring treatment adherence and providing effective emotional support. Another critical challenge is mental state modeling. A mental state is complicated, and the user's emotion intensity will subtly fluctuate during the whole conversation. Thereafter, the support strategy selection will differ depending on different mental states.

**Figure 2:**
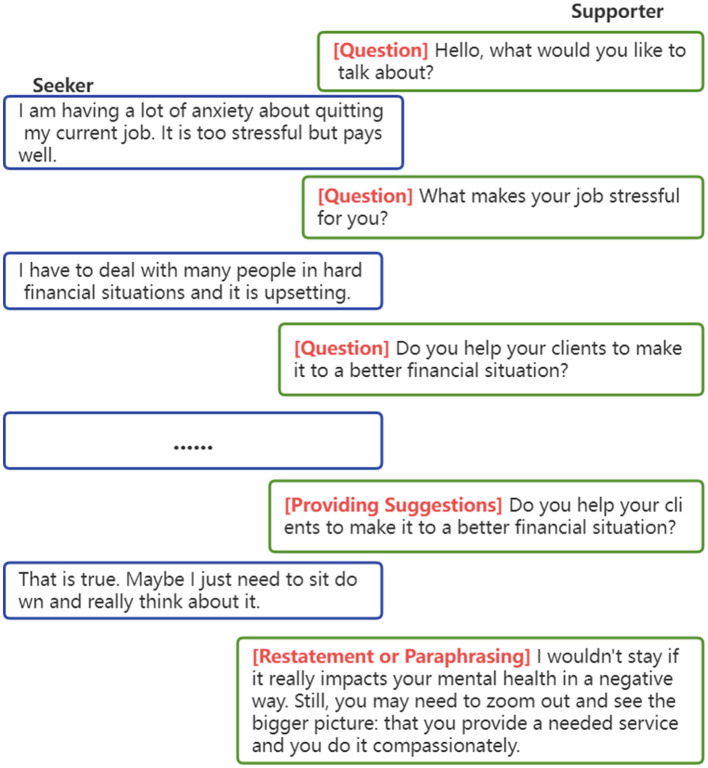
An example to show a multi-turn emotional support conversation between supporter and seeker. The red parts are strategies the supporter used.

Figure 2 shows a typical emotional support scenario. The supporter first strategically comforts the seeker by caringly enquiring about the problem, then resonating with the seeker's feelings, and then providing suggestions to evoke positive emotions. Due to the particularity of multi-turn dialog scenarios, the ESC system should further take into account how much the selected strategy will contribute to lessening the user's emotional suffering over time. Even though some strategies might not immediately contribute to offering emotional support, they are still effective for accomplishing the long-term goal.

To validate the performance of the ESC system, Liu et al. (30) also released ESConv, a dataset including 1,053 multi-turn dialogs with 31,410 utterances. ESConv contains eight kinds of support strategies to enhance the effectiveness of emotional support, which are questions, restatements or paraphrasing, reflection of feelings, self-disclosure, affirmations reassurance, and providing suggestions, information, and others, almost uniformly distributed among the whole dataset (30). Each example in the ESCconv dataset consists of the psychological problem of the seeker (situation), the whole process of dialog (utterances), and the skills the helper adopted (strategy).

However, how to evaluate the effectiveness of emotional support remains to be explored. Following Liu et al. (30) and Tu et al. (31), we also exploit automatic evaluation and human evaluation to evaluate our work, as described below.

#### 2.1.1. Automatic evaluation

To measure the diversity of responses in the conversation system and the performance of generated response, we adopted traditional evaluation methods PPL(perplexity), D-2(Distinct-2), BLEU(B-2, B-4) (35), and R-L (ROUGE-L) (36). In addition, we employed an extra metric, **ACC** (strategy prediction accuracy), mentioned in MISC (31) to indicate the ability to select an accurate strategy.

#### 2.1.2. Human evaluation

We adopted the questionnaire (30) mentioned. Thereafter, volunteers would be asked the following questions: (1) **Fluency:** Which of the following responses is more fluent and easier to understand? (2) **Identification:** Which answer is more accurate about your situation and more helpful in identifying your problem? (3) **Comforting:** Which answer makes you feel more comfortable? (4) **Suggestion:** Which answer between the two candidates gives advice that contains specific methods that are more useful to you than the other one? (5) **Overall:** Generally speaking, which of these two forms of emotional support do you prefer overall?

### 2.2. STEF agent

**Figure 3:**
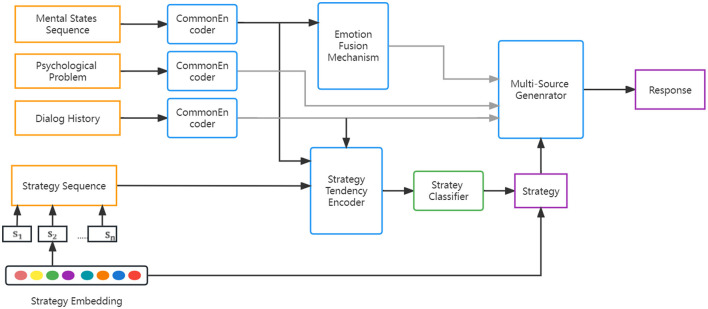
Architecture of STEF agent consists of a strategy tendency encoder, an emotional fusion mechanism, and a multi-source generator.

In Figure 3, the STEF agent consists of several primary components. The strategy tendency encoder employs historical strategies, dialog context, and historical mental states to capture the interaction and latent semantic embedding of strategy. The emotion fusion mechanism controls the fusion of the seeker's current mental state and past mental states. The multi-source generator generates a supportive response by considering multiple factors, including latent strategy embedding and the fusion information of the seeker's mental state.

#### 2.2.1. Preliminary work

Emotional support conversation is a generation task, and we can define this task as below. Given a sequence of utterances in dialog history $D=\left\{{\left(x_{i},y_{i}\right)}_{i=1}^{n-1}\right\}$, where *x*_*i*_, *y*_*i*_ are spoken by the seeker and the supporter, respectively, *i* denotes the index of round, and *n*−1 denotes the round number of history conversation. In addition to *D*, inputs for the ESC task also include historical strategy sequence *S* = {*s*_1_, *s*_2_, …, *s*_*n*−1_}, the seeker's last utterance with m words *B* = {*b*_1_, *b*_2_, …, *b*_*m*_}, and a psychological problem with p words *C* = {*c*_1_, *c*_2_, …, *c*_*p*_}. Hence, the goal of this task is to generate a supportive response conditioned on the dialog history *D*, history strategies *S*, the seeker's last utterance *B*, and the seeker's psychological problem *C*.

Blenderbot-small (37) is an open-domain conversation agent pretrained with multiple communication skills and large-scale dialog corpora. Blenderbot-small employs poly-encoder in the standard seq2seq transformer architecture. The poly-encoder utilizes a cross-encoder and multiple representations to encode features (38). Following the previous work (39, 40), we utilize the encoder of blenderbot-small as our common encoder to represent historical strategies. The representation of dialog history can be formulated as follows:


(1) $$\begin{array}{l} H_{D}=Enc(CLS,(x_{1},SEP,y_{1}),SEP,(x_{2},SEP,y_{2}),\ldots ,\\ \;(x_{n-1},SEP,y_{n-1})), \end{array}$$

where *Enc* is the encoder, *CLS* is the start token, and *SEP* is the separation token between two utterances.

In the ESC task, the supporter chooses a different strategy to comfort the seeker based on the seeker's different mental conditions, which indicates that the seeker's mental states are important. We exploit COMET (41) to capture the seeker's mental states. COMET, a commonsense knowledge generator, utilizes the natural language tuples (event, pre-defined relation) to generate corresponding knowledge. We consider each seeker's utterances in dialog history as an event and input each of them into COMET to acquire a collection of mental states.


(2) $$\begin{array}{l} \;M=\{emo_{1},emo_{2},\ldots ,emo_{n-1}\},\\ emo_{i}=\mathrm{COMET}\left(rel_{xAttr},x_{i}\right), \end{array}$$

where *M* is the sequence of user's mental states, *rel*_*xAttr*_ is one of the pre-defined relations in COMET, and *u*_*i*_ is the utterance of the seeker. The relation xAttr in COMET denotes how the person might be described in an event (utterances). Note that the outputs of *COMET* are a series of emotion-related synonyms, and we select the first result as ***emo*_*i*_**.

Furthermore, we also use our common encoder to represent the sequence of historical mental states obtained from COMET.


(3) $$\begin{array}{l} H_{e}=[h_{e_{1}},h_{e_{2}},\ldots ,h_{e_{n-1}}],\\ \;h_{e_{i}}=Enc(emo_{i}), \end{array}$$

where ***H*_*e*_** is the representation of historical mental states and ***h*_*e*_*i*__** is the hidden state of the encoder. Similarly, we can feed the seeker's last utterance to obtain the seeker's current mental state ***emo*_*B*_** using COMET. The representation ${{\text{H}}_{\text{e}}}^{\text{B}}$ will be obtained using a common encoder. Finally, we have representations of the seeker's mental state at the dialog and utterance levels.

According to Liu et al. (30), each conversation in ESConv has long turns, and they truncate them into pieces. Hence, the psychological problems of each conversation are critical to enhancing the understanding of conversation pieces. To derive the psychological problem's representation *H*_*g*_, we continue to employ the common encoder:


(4) $$H_{g}=Enc(C).$$

#### 2.2.2. Emotional fusion mechanism

Motivated by the study by Peng et al. (42), we propose an emotional fusion mechanism for effectively integrating mental state information from the whole conversation and acquiring the influence of historical emotion. The fusion layer is combined the representation of historical and current mental states. Our fusion kernel simply employs concatenation, addition, and subtraction operations to fuse the two sources. According to Peng et al. (43) and Mou et al. (44), it is effective to fuse different representations by utilizing a heuristic matching trick with a difference and element-wise product in the fusion mechanism. Hence, an emotional fusion mechanism can be formulated as


(5) $$\begin{array}{l} H_{e}=\mathrm{Fuse}(H_{e}^{u},H_{e}^{B})\\ \;Fuse\left(H_{e}^{u},H_{e}^{B}\right)=\mathrm{Relu}(w_{f}^{T}[H_{e}^{u};H_{e}^{B};H_{e}^{u}{}^{\circ}H_{e}^{B};H_{e}^{u}\\ \;+H_{e}^{B};H_{e}^{u}-H_{e}^{B}]+b_{f}) \end{array}$$

where *Fuse* is the fusion kernel, *Relu* is non-linear transformation, **◦** denotes the element-wise product, and the *w*_*f*_, *b*_*f*_ are learnable parameters.

#### 2.2.3. Strategy tendency encoder

It is essential that the ESC system chooses an appropriate strategy based on the seeker's mental states and generates a strategy-constrained response. Inspired by DialogEIN (45), we propose the strategy tendency encoder to capture the tendency of each utterance and the latent strategy information. As shown in Figure 3, the embedding of each category is depicted by the circles with different colors. Given the set of strategy labels T= {*t*_1_, *t*_2_, …, *t*_*q*_}, each strategy embedding can be formulated as


(6) $$e_{i}=E^{t}(t_{i}),$$

where *E*^*t*^ denotes the strategy embedding lookup table and *e*^*i*^ indicates the embedding of the *i*-th strategy category. We initialize the strategy embedding randomly and tune them during the model training. The dimension of strategy embedding is the same as the representations of dialog history and mental states for exploring the interaction from them. Thereafter, we use the strategy embedding to construct the representation of history strategies, *S*, denoted as


(7) $$E_{s}=[e_{s_{1}},e_{s_{2}},\ldots ,e_{s_{n-1}}],$$

where *e*_*s*_*i*__ denotes the history strategy embedding for the *i*-th utterance.

To capture the evolution of a support strategy, a multi-head attention module is applied. Based on DialogEIN, we modify the multi-head attention module as


(8) $$H_{s}=\mathrm{MHA}(H_{D},H_{e},E_{s})+H_{D},$$

where MHA stands for the multi-head attention module, *H*_*D*_ is the query, *H*_*e*_ is the key, and *E*_*s*_ is the value of the self-attention mechanism. *H*_*s*_ indicates the tendency information of strategy explicitly and contains the interaction information of historical strategies and mental states. We add the residual of query *H*_*D*_ to *H*_*s*_ to ensure it sustains semantic information.

Thereafter, we train a multi-class classifier to predict the response strategy distribution for fully using strategy tendency information *H*_*s*_. By combining the distribution and strategy embedding, we derive latent strategy representation $H_{s}^{\prime }$ as follows:


(9) $$\begin{array}{l} s_{p}=\text{multi}-\text{classifier}(H_{s}),\\ H_{s}^{\prime }=s_{p}*[e_{1},e_{2},\ldots ,e_{q}], \end{array}$$

where multi-classifier is a multi-layer perceptron, *s*_*p*_ is the strategy probability distribution prediction, and [e_1_, e_2_, …, e_q_] is the embedding set of the strategy label.

#### 2.2.4. Multi-source generator

For conversational agents, the decoder learns a continuous space representation of a phrase that preserves both the semantic and syntactic structure of the utterance. To generate a supportive response, we fully integrate all kinds of information from the above-mentioned source. In MISC (31), the cross-attention module of the blenderbot-small decoder is modified to utilize the strategy representation and mental states. We retain this module and employ multi-source representation in our model to obtain cross-attention.


(10) $$\begin{array}{l} A_{d}=Cross-attn(O,H_{D}),\\ A_{s}=Cross-attn(O,H_{s}^{'}),\\ A_{e}=Cross-attn(O,H_{e}),\\ A_{g}=Cross-attn(O,H_{g}), \end{array}$$

where O is the hidden states of the decoder and *Cross* − *attn* is the cross-attention module.

##### 2.2.4.1. Loss function

The architecture of our model has two tasks: predict the strategy and generate the response. In this study, we directly adopted the same objective from MISC to train our model.


(11) $$\begin{array}{l} L_{r}=-\sum_{t=1}^{n_{r}}log(p(r_{t}|r_{j<t},D,M,C,S)),\\ L_{s}=-log(p(s^{'}|D,M,C,S)),\\ L=L_{r}+L_{s}, \end{array}$$

where *L*_*r*_ is the loss of generated response, *n*_*r*_ is the length of generated response, *L*_*s*_ is the loss of predicting strategy label, *s*′ is the ground truth of the strategy label, and *L* is the combined objective to minimize.

### 2.3. Procedures

Our experiments were conducted on the ESConv dataset, following the MISC division of the ESconv dataset for 9882/1235/1235 samples for the training, validation, and testing of partitions. We fine-tuned STEF agent based on the blender-bot small with the size of 90M parameters. The maximum length of the input sequence for the common encoder is 512, and the dimension of all hidden embeddings is 512. We set the training batch size and evaluating batch size to 8 and 16, respectively, to fit GPU memory and the dropout rate to 0.1. Following the previous work, we employed linear warm-up in 120 warm-up steps. We also employed AdamW as an optimizer, which builds upon the Adam optimizer and incorporates weight decay to improve the performance of regularization. The number of epochs (10 to 40) and initial learning rate (5e-4 to 5e-6) were also tuned. We evaluated perplexity for each checkpoint on the validation set, finally selecting the one corresponding to the lowest perplexity as the trained model. We used one GPU of the NVIDIA Tesla V100 to train the STEF agent, and the overall training time was 1.5 h. During training, we observed that the STEF agent trained for 20 epochs with a learning rate of 2e-5 showed the best performance based on perplexity.

After training, we evaluated the model on the test dataset through two dimensions: automatic evaluation and human evaluation. In automatic evaluation, our model was compared to the baseline in terms of the accuracy of the predicted strategy and common LM metrics of generated responses. In human evaluation, we recruited 10 annotators and asked them to complete questionnaires. Each questionnaire includes two responses generated by our model and another model separately. The annotator compared the two responses on five aspects (fluency, identification, comforting, suggestion, and overall) and annotated the better one. A total of 64 samples were selected from the test set for response generation, and two other models were compared to ours.

## 3. Analysis

### 3.1. Experiment results

#### 3.1.1. Automatic evaluation

We compared our model with several baseline models: Transformer, MoEL (46), MIME (25), Blenderbot-joint (30), and MISC (31), GLHG (32). The metric of perplexity (PPL) measures the quality of generated responses from the language model dimension, indicating that it is more capable of producing high-quality responses. Distinct-2 (D-2) measures the ratios of the unique 2 g in the generated response. BLEU-n (B-2, B-4) measures the ratios of the common n-gram token number between generated and ground-truth responses to the length of the generated response. Rouge-L (R-L) measures the longest common sub-sequence between the generated and ground-truth responses. In Table 1, the STEF agent has a promising result on D-2 compared with baseline models. This result demonstrates that the response the STEF agent generated is more diverse than other baselines. The conversational agent in the DTx solution focuses on personalization and customization, which means that the agent should generate diverse responses. Hence, the D-2 result can also demonstrate that the STEF agent is appropriate for the DTx solution. In terms of the Rouge-L metric, we can see that the Rouge-L result outperforms most baselines, including Blenderbot-joint and GLHG. The Rouge-L result demonstrates that the STEF agent can mimic a supporter to show understanding and comfort the seekers. By comparing with the SOTA models Blenderbot-Join and MISC, we can see that the STEF agent has the worse performance for the Acc metric and perplexity. However, the support strategy is an alternative, and other strategies may also have an effect; thus, the accuracy (ACC) metric is insufficient to evaluate the strategy. The comparison results demonstrate that the STEF agent has the potential to be applied to the DTx product.

**Table 1:** Results of automatic evaluation.

**Model**	**ACC↑**	**PPL↓**	***D*−2↑**	***B*−2↑**	***B*−4↑**	***R*−L↑**
Transformer	−	89.61	6.91	6.53	1.37	15.17
MoEL	−	133.13	15.26	5.93	1.22	14.65
MIME	−	47.51	10.94	5.23	1.17	14.74
BlenderBot-Joint	28.57	18.49	17.72	5.78	1.74	16.39
MISC	31.63	16.16	19.71	7.31	2.20	17.91
GLHG	−	15.67	21.61	7.57	1.03	16.37
STEF(Ours)	**25.70**	**18.42**	**23.00**	**6.96**	**1.58**	**16.40**

Bold values indicate that ACC: The strategy prediction accuracy. PPL: Perlexity. PPL measures the quality of generated responses from the language model dimension. D-2: Distinct-2 (D-2) measures the ratios of the unique two-grams in the generated response. The format is count (two-gram) / count(word). B-2, B-4: Bleu-2, Bleu-4 from Bleu-n. The bleu-n measures the ratios of the common n-gram token number between generated and ground-truth responses to the length of the generated response. R-L: Rouge-L (R-L) measures the longest common sub-sequence between the generated and ground-truth responses. Win: When the volunteers thought the generated response was superior to the other response, they labeled the sample “Win”. Lose: When the volunteers thought the generated response was inferior to the other response, they labeled the sample “Lose”. Tie: When the volunteers thought the generated response was equal to the other response, they labeled the sample “Tie”.

#### 3.1.2. Human evaluation

As above mentioned in the procedure, we recruited 10 volunteers to complete the questionnaire. To assist the volunteer in acting as the support seeker as effectively as possible, each sample in the questionnaire includes information on a mental problem description and dialog history. The volunteer was asked to label the generated response with the “win" label when they thought the generated response was superior to the other response. At last, we made a statistical analysis of these questionnaires from three aspects (win, lose, and tie). The human evaluation results in Table 2 show that our model has a substantial advantage in all aspects. Compared to Blenderbot-joint, our model significantly outperforms the comforting and suggestion aspect, which indicates that our model is capable of showing support and providing suggestions better. However, in terms of the fluency aspect, our model does not gain much. Compared to MIME, our model achieves remarkable advancement on all metrics, especially on the fluency and identification aspect, which demonstrates our model's best ability to provide emotional support.

**Table 2:** Performance of human evaluation (%).

**Comparisons**	**Aspects**	**Win**	**Lose**	**Tie**
STEF(Ours) vs. BlenderBot-Joint	Fluency	**44.6**	35.9	19.5
	Identification	**49.2**	30.1	20.7
	Comforting.	**60.7**	22.4	16.9
	Suggestion	**57.1**	27.8	15.1
	Overall	**56.4**	28.3	15.3
STEF(Ours) vs. MIME	Fluency	**63.4**	22.7	13.9
	Identification	**66.5**	21.9	11.6
	Comforting	**53.2**	32.1	14.7
	Suggestion	**55.8**	26.3	17.9
	Overall	**60.3**	22.5	17.2

Bold values indicate that ACC: The strategy prediction accuracy. PPL: Perlexity. PPL measures the quality of generated responses from the language model dimension. D-2: Distinct-2 (D-2) measures the ratios of the unique two-grams in the generated response. The format is count (two-gram) / count(word). B-2, B-4: Bleu-2, Bleu-4 from Bleu-n. The bleu-n measures the ratios of the common n-gram token number between generated and ground-truth responses to the length of the generated response. R-L: Rouge-L (R-L) measures the longest common sub-sequence between the generated and ground-truth responses. Win: When the volunteers thought the generated response was superior to the other response, they labeled the sample “Win”. Lose: When the volunteers thought the generated response was inferior to the other response, they labeled the sample “Lose”. Tie: When the volunteers thought the generated response was equal to the other response, they labeled the sample “Tie”.

The automatic evaluation result and human evaluation result reveal that the STEF agent has a promising performance of support though the **ACC** metric of strategy prediction is lower than the competitor baseline. The seeker's mental states affect the selection of support strategies. Even when faced with a small mental problem, the seeker's mental state still changed with different support strategies. The support strategy is an alternative in different ESC stages; consequently, the ground truth of the support strategy is not unique. The human evaluation results can demonstrate that the strategy tendency encoder can construct the strategy evolution for the whole conversation and predict the appropriate support strategy to improve the performance.

### 3.2. Case study

Table 3 presents two cases to illustrate the effectiveness of our model. We can see that the situations in cases 1 and 2 are consistent. The seekers both faced depression caused by COVID-19, which has been prevalent around the world in recent years. In these cases, the conversational agent of DTx product in the mental area has the advantage of identifying the situation seeker encountered timely. Conversational agents with the ability of emotional support could accompany the seeker using strategy and provide suggestions to alleviate the mental problem.

**Table 3:** Examples in the test set of ESConv.

**Situation: I am depressed staying home due to COVID-19. (Case 1)**
**Supporter**: Have you been worrying about anything?
**Seeker**: I have been staying home since last March due to COVID-19; getting very depressed.
**Supporter**: Ah I hear you, I have been self isolating too. Do you have an online support network?
**Seeker**: What do you mean by support network?
**Supporter**: Well, friends you can talk to during this difficult time? I have a lot of friends on Facebook and similar that I can talk to.
**Seeker**: I have few friends and can talk to but not sure how would they help.
**Ground-Truth**: Sometimes it does just make it easier to know that other people are in the same situation as you :
**MISC**: I know how it feels! you will come out of this year with a stronger person.
**Our model**: I'm sorry to hear that, but talking with a friend can help you feel better.
**Situation: I lost my job Due to COVID-19. (Case 2)**
**Supporter**: Hello! How may I assist you today?
**Seeker**: Hey, Mmm I haven't slept.
**Supporter**: It sounds you did not sleep. am I right?
**Seeker**: I lost my job due to COVID-19 , Now I not able to satisfy my needs.
**Supporter**: Oh! Sorry to hear about that. I can understand it might be tough for you to deal all this.
**Seeker**: I asked help from my friend, relatives and applied in online. Not yet receive any positive result.
**Ground-Truth**: I know you can handle this tough time situation. These tough time teach us so many lessons and give the solutions too.
**MISC**: I've been in a similar situation. I know it is difficult, but you can get one.
**Our model**: I know it's hard but I'm sure everything's gonna be okay.

The situation is the mental problem seeker faced, the middle part is dialog history, and the lower part has generated responses from MISC and our model.

In **Case 1**, our model first expresses its understanding of the seeker's description and then explains the benefits of talking with a friend. The response of our model is better than MISC's response, as shown in Table 3. The latter part of the response MISC generated just encouraged the seeker neutrally without understanding historical support strategies despite the fact that the MISC's response also initially demonstrated comprehension. Compared to MISC, our model can appropriately comfort the seeker according to the historical support strategies.

In **Case 2**, our model first affirms that the problem the seeker faces is difficult and provides the seeker with empathetic encouragement. Compared to the response our model generated, the MISC's response is not reasonable in this scenario, particularly in the DTx area. Users of DTx products are aware the conversational agent will be with them 24/7; hence, the expression will make them feel ridiculous, reducing the reliability of DTX products even further.

The two cases above have also been evaluated by annotators, and their feedback demonstrates that our model is able to comprehend the seeker's mental state, choose an appropriate strategy, and generate a supportive response. Compared to the ground truth, the generated responses of our model have the same effect on the seeker.

The cases demonstrate that the STEF agent can show understanding and comfort the users. The STEF agent can be employed in the DTx solution to provide a personalized response based on the various symptoms of patients. The strategy of the STEF agent can be replaced or supplemented with more professional mental counseling skills. Thereafter, the STEF agent in the DTx platform can utilize recorded dialog to be more helpful and professional. Furthermore, the STEF agent can utilize the translation technologies to provide multilingual service for patients from all over the world.

## 4. Conclusion

This paper proposes a novel conversational agent with a concentration on historical support strategies and the fusion of the seeker's mental states. We proposed the strategy tendency encoder to obtain the tendency of support strategies and the emotional fusion mechanism to gain the influence of historical mental states. Experiments and analysis demonstrate that the STEF agent achieves promising performance. However, we find that our results tend to include content that commonly appears in many samples (e.g., “I'm sorry to hear that,” “I'm glad to hear that,” “I understand”). The results show a lack of diversity and are unable to show personalization, which is insufficient in ESC. There are other limitations, including those as follows: (1) The available data are inadequate, and the support strategy must be annotated. It is costly to train crowd workers to annotate the vast amount of data. (2) The support strategy should be more alternative in each phase. How to evaluate whether the strategy is appropriate is worth exploring. For future studies, we plan to improve the STEF agent based on the above limitations.

## Data availability statement

The original contributions presented in the study are included in the article/supplementary material, further inquiries can be directed to the corresponding authors.

## Author contributions

QW developed the conversational agent, conducted the experiment, analyzed the data, created all figures, and wrote the manuscript. SP contributed to the research by providing critical feedback and editing the manuscript. ZZ created all figures and also conducted experiments. XH supervised the entire research process and providing guidance throughout. CD, LH, and PH contributed to the research by providing critical feedback. All authors contributed to the article and approved the submitted version.
